# The Magazine of Botany and Gardening, British and Foreign

**Published:** 1834-07-01

**Authors:** 


*The Magazine of Botany and Gardening, British and Foreign.* Edited by JAMES RENNIE, M.A., Professor of Zoology, King's College. London, 1834.

THIS is one of those dishonest publications against which, notwithstanding their cheapness and occasional value, we resolutely set our face; for the injury they do to the commonwealth of science is incalculable. Like smugglers, or pirates, or the dealers in stolen goods, they offer the wares pilfered from their neighbours at a much lower price than they could be manufactured by the honest literary operative; as that which costs nothing may be sold for nought. But it behoves authors and publishers to withstand the parasites which not only feed upon their labours, but undersell them in the markets with their own goods.

In looking over several of the late numbers of the Magazine whose editor's name —graces the head of this notice, we have found scarcely anything original in them, excepting errors which have been ignorantly introduced, or negligently suffered to escape correction. If anything can exceed the assurance with which articles are filched from every side, it is the calm effrontery with which the announcements are worded, so as to lead the public to believe that the persons who have been robbed have voluntarily contributed their property. We fear that our language is suited rather to our subject than to our own pages; but we know not any terms of condemnation sufficiently severe to characterise such a system of plunder; and, although modern refinement may use a smoother name, it seems to us that illegal conveyancing does not deserve it.

